# Structure of CbpA J-Domain Bound to the Regulatory Protein CbpM Explains Its Specificity and Suggests Evolutionary Link between CbpM and Transcriptional Regulators

**DOI:** 10.1371/journal.pone.0100441

**Published:** 2014-06-19

**Authors:** Naghmeh S. Sarraf, Rong Shi, Laura McDonald, Jason Baardsnes, Linhua Zhang, Miroslaw Cygler, Irena Ekiel

**Affiliations:** 1 Department of Chemistry and Biochemistry, Concordia University, Montréal, Québec, Canada; 2 Life Sciences, National Research Council of Canada, Montréal, Québec, Canada; 3 Département de biochimie, de microbiologie et de bio-informatique, et L'Institut de biologie intégrative et des systèmes, et PROTEO, Université Laval, Québec City, Québec, Canada; 4 Department of Biochemistry, McGill University, Montréal, Québec, Canada; 5 Department of Biochemistry, University of Saskatchewan, Saskatoon, Saskatchewan, Canada; University of South Florida College of Medicine, United States of America

## Abstract

CbpA is one of the six *E. coli* DnaJ/Hsp40 homologues of DnaK co-chaperones and the only one that is additionally regulated by a small protein CbpM, conserved in γ-proteobacteria. CbpM inhibits the co-chaperone and DNA binding activities of CbpA. This regulatory function of CbpM is accomplished through reversible interaction with the N-terminal J-domain of CbpA, which is essential for the interaction with DnaK. CbpM is highly specific for CbpA and does not bind DnaJ despite the high degree of structural and functional similarity between the J-domains of CbpA and DnaJ. Here we report the crystal structure of the complex of CbpM with the J-domain of CbpA. CbpM forms dimers and the J-domain of CbpA interacts with both CbpM subunits. The CbpM-binding surface of CbpA is highly overlapping with the CbpA interface for DnaK, providing a competitive model for regulation through forming mutually exclusive complexes. The structure also provides the explanation for the strict specificity of CbpM for CbpA, which we confirmed by making mutants of DnaJ that became regulated by CbpM. Interestingly, the structure of CbpM reveals a striking similarity to members of the MerR family of transcriptional regulators, suggesting an evolutionary connection between the functionally distinct bacterial co-chaperone regulator CbpM and the transcription regulator HspR.

## Introduction

In response to environmental stress, including heat, all cells produce heat shock proteins (HSPs), the most important classes of which include chaperones and proteases. Many heat shock proteins are among the most conserved proteins known; however, they possess diverse regulatory mechanisms. Interestingly, in bacteria, HSP chaperones are often directly involved in regulation of transcription through interactions with transcription regulators or sigma factors [1]–[3]. This contribution can be either positive (e.g. involving sigma32 factors), or negative (e.g. involving transcription regulators such as HspR and HrcA) [1].

Molecular chaperones of the Hsp70 class bind and stabilize proteins at intermediate stages of folding, degradation, assembly and translocation across membranes. They are required for growth at normal temperatures, but their level of expression is enhanced under conditions of stress. The most important for bacterial viability and the most extensively characterized Hsp70 chaperone in *Escherichia coli* is DnaK [4]. The activity of DnaK/Hsp70 chaperones is regulated by co-chaperones, members of the DnaJ/Hsp40 family [5]–[8]. DnaJ is composed of four domains. The N-terminal strongly conserved ∼70 residue long so called J-domain [9], the central cysteine-rich domain and two C-terminal domains of similar fold. DnaJ stimulates ATPase activity of DnaK, through conformational change in DnaK from the ATP-bound state, which binds substrates weakly, to an ADP-bound state, which binds substrates tightly [4]–[7]. Biochemical studies on the *E. coli* DnaK–DnaJ system have shown that the J-domain, and in particular its H-P-D sequence motif, is important for both DnaK binding and ATPase stimulation [10], [11]. These processes are mediated by direct interaction between the J-domain of a co-chaperone and the ATPase domain of DnaK/Hsp70 [7]. J-domains and their mechanism of chaperone regulation are highly conserved from bacteria to humans [8].

There are six known co-chaperones of the DnaJ/Hsp40 family in *E. coli*, three of which, DnaJ, CbpA (cytosolic proteins) and DjlA (membrane associated), bind to DnaK [7]. DnaJ has been identified as a key regulator of various DnaK activities [6], [7], [10]. CbpA constitutes a functional homolog of DnaJ [12] and overexpression of CbpA can complement for all known phenotypes associated with the loss of DnaJ [13]–[15]. CbpA was originally isolated by virtue of its retention on an intrinsically curved DNA affinity column and named “curved DNA binding protein A” [13]. It is a major protein associated with *E. coli* nucleoids in stationary growth [16] but the function of its DNA-binding activity, differentiating the two co-chaperones, is just starting to be explored [14], [17]–[19].

Among the three Hsp40 proteins that function as DnaK co-chaperone, only CbpA is regulated through interaction with a specific partner protein, CbpM [15]. The biological processes regulated by CbpM are only beginning to be understood [20]. Its gene lies downstream of *cbpA* within the same operon and is homologous to proteins encoded by genes located downstream of *dnaJ*-like genes in a diverse range of bacteria. It has been shown that CbpM inhibits both CbpA co-chaperone activity and its DNA binding [14]. It has been suggested that during certain growth phase or stress conditions, CbpA might be released from CbpM and recruit DnaK to function as a co-chaperone [15], [21].

We proposed recently that CbpM competes with the DnaK chaperone for CbpA, providing a plausible mechanism of regulation [22]. We have utilized NMR and site-directed mutagenesis to characterize the CbpA^Jdom^ surface that forms the interface for the CbpM [22]. Here we expand our understanding of the chaperon regulation by determining the crystal structure of the complex of CbpA^Jdom^ with CbpM, which defines the structural basis of this interaction, explains the specificity of CbpM for CbpA *vs* DnaJ, and clarifies the mechanism by which CbpM inhibits CbpA co-chaperone activity.

Unexpectedly, we observed that CbpM displays striking structural similarity to MerR-like transcription regulators and at the same time an architectural difference, which reflects different function of these two groups of proteins. The structural similarity suggests the evolution of function of an ancient protein family from transcription regulation to chaperone system regulation and we propose a mechanistic model explaining such a transition.

## Materials and Methods

### Plasmids and Bacterial Strains

Constructs expressing CbpA^Jdom^ were prepared by generating a PCR fragment coding for CbpA (residues 2–73 or 2–76) from *E. coli*-K12 genomic DNA, which were ligated into the expression vector pFO1, a derivative of pET15b (Novagen), to obtain an N-terminal His_8_-tagged thrombin-cleavable construct. The CbpM PCR fragment (residues 2–101) amplified from *Klebsiella pneumoniae* (strain ATCC 700721/MGH 78578) and *E. coli* K-12 genomic DNA as well as full length *E. coli* CbpA were ligated into pJW271, a derivative of the pMAL-c2X vector (New England BioLabs Inc.) to obtain a TEV-protease cleavable, N-terminal His-MBP-fusion protein. *E. coli* DnaK, DnaJ and DnaJ^Jdom^ (residues 2–79) were expressed from pRL652, a derivative of pGEX-4T1 vector (GE Healthcare) to obtain N-terminal GST-fusion constructs with TEV cleavage site. After verification by DNA sequencing, the constructs were transformed into *E. coli* Rosetta pLysS (Novagen) for protein expression. Site directed mutagenesis was done with the QuikChange Site-Directed Mutagenesis kit (Stratagene) as recommended by the manufacturer.

### Protein Expression and Purification

All proteins were expressed in Luria-Bertani (LB) media at 22°C for 18 hrs. Recombinant CbpA, CbpM and CbpA^Jdom^ were purified by standard immobilized metal affinity chromatography using Ni^2+^-NTA resin (Qiagen) using 50 mM TRIS-HCl, pH 8.0 with 250–300 mM NaCl and eluted with the same buffer containing 200–350 mM imidazole. DnaK, DnaJ and DnaJ^Jdom^ were purified using the glutathione sepharose resin (GE Healthcare). The tags were cleaved using either thrombin or tobacco-etch virus protease (TEV), depending on the construct. Following cleavage, the tag was removed from the protein sample using Ni-NTA resin (New England BioLabs Inc). For CbpM and CbpA, the protein samples were also passed through amylose resin (New England Biolabs Inc). All proteins were further purified using size exclusion. For crystallization, the complex CbpM-CbpA^Jdom^ was loaded on a Superdex 75 column and separated from excess CbpA^Jdom^. For ATPase assays, the proteins were purified on a Superdex 200 column equilibrated with 20 mM Tris pH 8, 150 mM NaCl, 1 mM MgCl_2_ and 0.2 mM DTT. Magnesium was omitted for CbpM to avoid contaminated ATPase that co-eluted in the presence of MgCl_2_. No ATPase activity for the CbpM sample was seen when MgCl_2_ was removed from the gel filtration buffer.

*ATPase assays* were performed using malachite PiColorlock ALS Phosphate detection system (Innova Biosciences), a malachite green based dye. For phosphate detection, an equal volume of ALS mix was added to samples and absorbance at 635 nm was measured after 30 minutes (Spectromax 250 plate reader). Phosphate release was linear in the presence of DnaJ and CbpA over the time point studied.

### Surface Plasmon Resonance Assays

All SPR assays were carried out using a ProteOn XPR36 instrument (Bio-Rad Laboratories Ltd., Mississauga, Ontario) with running buffer containing 50 mM sodium phosphate buffer (pH 7.4), 100 mM NaCl and 1 mM DTT, with the addition of 0.005% (v/v) Tween 20. All the experiments were performed using freshly immobilized CbpM under the same conditions, including immobilization, as described before for CbpM/CbpA^Jdom^ interactions [22].

Three running buffer-blank injections preceded each replicate series of analyte injections in order to stabilize the baseline. Each analyte was injected at a flow rate of 50 µL/min for 120 s at 25°C. Each set of analyte sensorgrams was double-referenced using the inter-spot reference and buffer blank analyte injection, and, where applicable, the steady-state K_D_ values were determined using the ProteOn Manager v2.1 software. The plateau RU values were used to generate binding isotherms, and the K_D_ was determined using the equilibrium fit (one site ligand).

### Crystallization

Crystals of the CbpM-CbpA^Jdom^ complex were obtained using the Protein Complex Suite screen (Qiagen). The best crystals were by hanging drop vapor diffusion by equilibrating 1µL of protein (15 mg/mL) with 1 µL reservoir solution (0.1 M HEPES pH 7, 20% (w/v) PEG 8K) over 0.5 mL of reservoir solution. The plates were set at 19°C, for 6 days, and then moved to 4°C, with crystals appearing after one week.

For structure determination, a native crystal was soaked for 1 min in reservoir solution supplemented with 0.4 M NaBr, 12% (v/v) ethylene glycol and flash cooled in the N_2_ cold stream (Oxford Cryosystem, Oxford, UK). Other native crystals were flash cooled using the reservoir solution supplemented with 12% (v/v) ethylene glycol as cryoprotectant.

### Data Collection and Refinement

Data collection was carried out at the CMCF1 beamline, Canadian Light Source. The Br-SAD data were collected to 2.6 Å resolution at a wavelength of 0.9197 Å. Crystals belong to space group *P*2_1_2_1_2_1_ with its unit cell dimensions *a* = 52.6, *b* = 74.0, *c* = 113.2 Å. Native diffraction data were collected to 1.87 Å at a wavelength of 0.9795 Å with unit cell *a* = 52.8, *b* = 77.1, *c* = 111.2 Å. Data processing and scaling were performed with HKL2000 [23]. The 18-site Br- substructure was determined with autoSHARP [24]. Solvent flattening with RESOLVE [25] led to phases with a figure-of-merit of 0.70 and automated model-building completed 75% of the expected residues in the asymmetric unit. The resulting model was then used for molecular replacement with MolRep [26] using the higher resolution native dataset, non-isomorphous to the Br-soaked crystal. Several cycles of refinement using REFMAC5 [27] followed by model rebuilding with Coot [28] were carried out, resulting in the final model with R_work_ = 0.195, and R_free_ = 0.230 (Table 1).

**Table 1:** X-ray data collection and refinement statistics.

Data set	Br-soaked (SAD)	CbpM-CbpA(Jdom)
Space group	*P*2_1_2_1_2_1_	*P*2_1_2_1_2_1_
a, b, c (Å)	52.6, 74.0, 113.2	52.8, 77.1, 111.2
wavelength(Å)	0.9197	0.9795
resolution (Å)	50–2.50 (2.59–2.50)	50–1.87 (1.94–1.87)
observed *hkl*	162,723	309,898
unique *hkl*	29,765^a^	38,222
redundancy	5.5 (5.1)	8.1 (7.6)
completeness (%)	100.0 (99.9)	99.7 (98.7)
^R^sym^b^	0.116 (0.497)	0.062 (0.489)
I/(σI)	20.0 (4.1)	30.8 (4.2)
Wilson B (Å^2^)	38.3	28.2
R*_work_* ^c^ (# *hkl*)		0.196 (36253)
R_free_ (# hkl)		0.232 (1911)
B-factors (# atoms)		
protein		31.0 (2814)
solvent		35.5 (450)
ligands		
Ramachandran		
allowed (%)		100%
generous (%)		0
disallowed (%)		0
rmsd's		
bonds (Å)		0.012
angles (°)		1.15
PDB code		3UCS

a Friedel pairs were unmerged.

^b^R_sym_^Σ^ = (|I_obs_−I_avg_^Σ^|)/I_avg_.

cR_work_^Σ^ = (|F_obs_−F_calc_^Σ^|)/F_obs_.

### Accession Numbers

Coordinates and structure factors have been deposited with the RCSB PDB under accession code 3UCS.

## Results

### Overall Structure of the CbpM-CbpA^jdom^ Complex

Initial attempts to obtain crystals of the *E. coli* CbpM-CbpA^Jdom^ complex were unsuccessful and as a result we expressed and purified protein orthologues from *Klebsiella pneumoniae* that show high sequence identity to their *E. coli* counterparts (71% and 89% identity for CbpM and CbpA^Jdom^, respectively). While this complex also resisted crystallization, a mixed complex containing *K. pneumoniae* CbpM and *E. coli* CbpA^Jdom^ led to well-diffracting crystals. This heterologous complex behaved better during purification and eluted as a single peak from size exclusion column.

**Figure 1:**
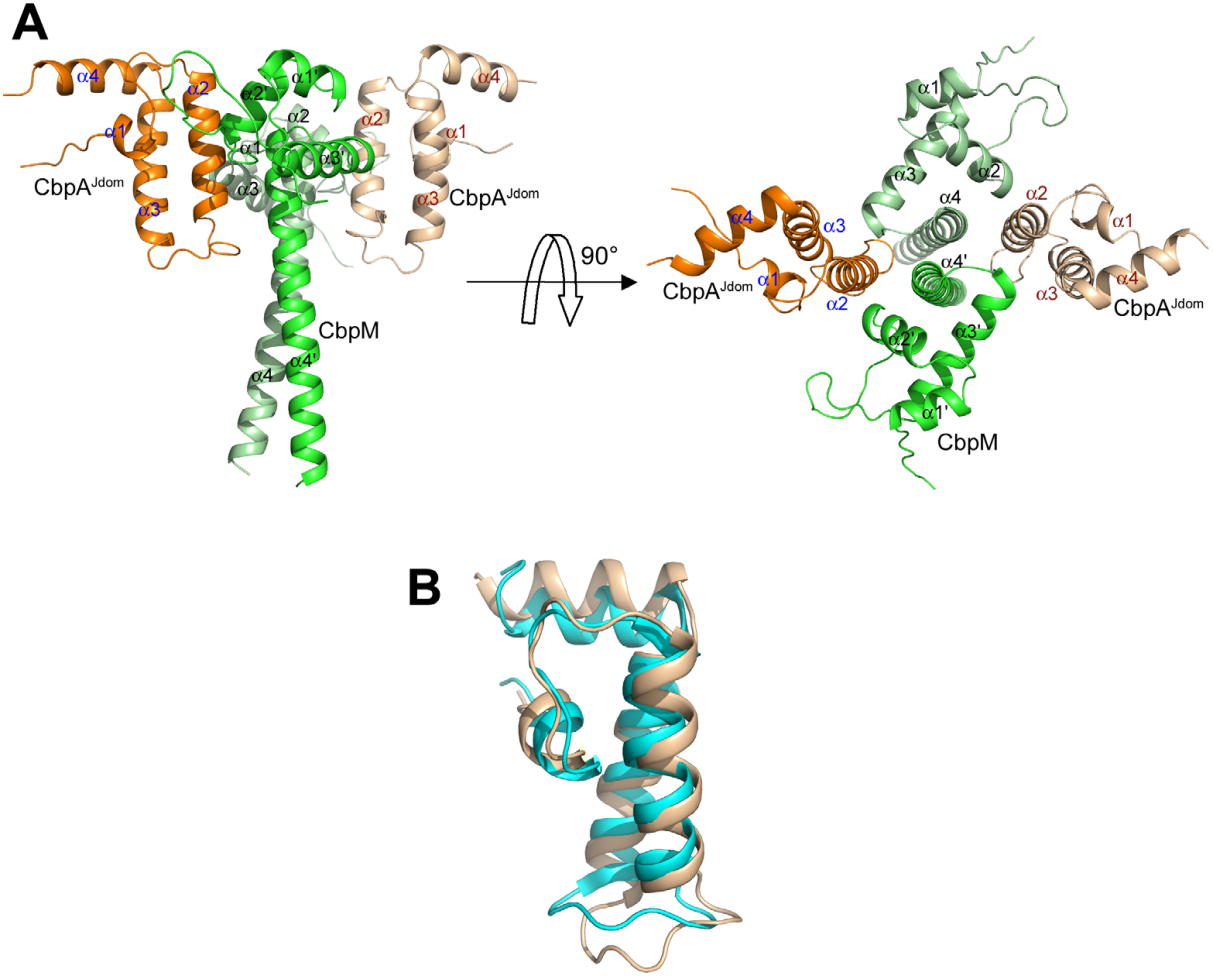
Structure of CbpM-CbpA^Jdom^. **A)** Overall structure of the complex. The CbpM dimers are in light and lime green and the two monomers of CbpA^Jdom^ are colored in orange and beige. The two CbpA^Jdom^ subunits on the opposite sides of the CbpM dimer are separated by approximately 20 Å; **B)** comparison of the NMR (cyan) and crystal structure of CbpA^Jdom^ (beige). Small conformational differences reside in the loop region. This and other figures were prepared with PyMol (http://www.pymol.org/).

The crystals of the CbpA^Jdom^-CbpM complex contain two CbpM and two CbpA^Jdom^ molecules in the asymmetric unit. The CbpM forms a tight dimer and the CbpA^Jdom^ subunits are bound to this dimer on opposite sides, near the globular domains (Fig. 1A). Thus CbpM and CbpA^Jdom^ form a heterotetramer with a 2∶2 stoichiometry. Size exclusion chromatography and dynamic light scattering indicate that a heterotetramer is also present in solution, strongly suggesting that this is the biological unit. Upon complex formation, each CbpA^Jdom^ molecule buries ∼740 Å^2^ or 13.5% of its total surface (∼5,480 Å^2^) while the buried surface of the CbpM dimer is ∼700 Å^2^ or 5.5% of the total solvent-accessible surface (12,700 Å^2^).

### CbpM Structure

The structure of *K. pneumoniae* CbpM is the first reported structure of a bacterial co-chaperone regulator. Each subunit of the CbpM dimer contains an N-terminal globular domain comprising three α-helices (residues 2–63) followed by a nine-turn helix, α4 (residues 67–99) that extends away from the globular domain (Fig. 1A). The dimer is formed through a parallel coiled-coil of the helix α4 with its α4′ counterpart from the other subunit. The parallel arrangement of coiled-coils in CbpM is stabilized by the presence of two asparagines (Asn80 and Asn87) in the a positions in the heptad repeats. Asparagines present in these positions are known to influence the helix orientation [29].

*CbpA^Jd^* ^o*m*^. The structure of CbpA^Jdom^ is similar to that previously determined by NMR [22] (PDB 2KQX) with four helices arranged as an orthogonal bundle, although the NMR model is more compact along the length of the bundle (Fig. 1B). The loop between helices α2 and α3 is usually structurally flexible in J-domains as shown by several NMR structures [22], [30]–[32]. We observe that in the CbpM-CbpA^Jdom^ complex structure, the N-terminal part of this loop participates in binding of CbpM and, in contrast to the free J-domain, this loop assumes a well-defined conformation.

### Molecular Interactions at the CbpA^jdom^ and CbpM Interface

**Figure 2:**
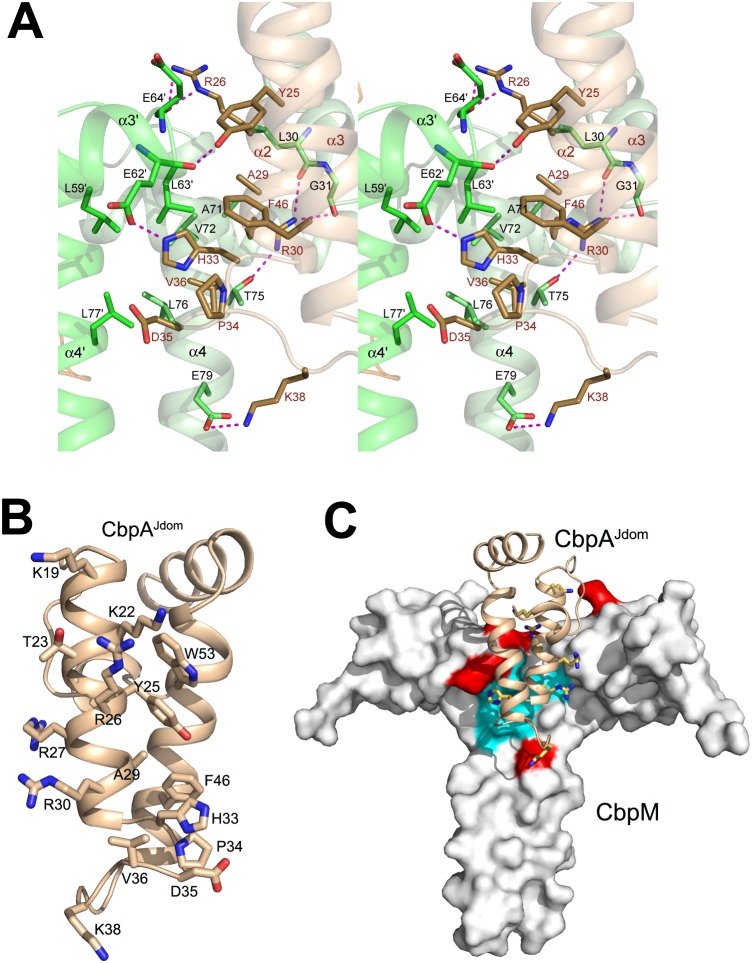
Interactions between CbpA and CbpM. **A)** The interacting residues of CbpM (in green) and CbpA^Jdom^ (in beige) on the complex interface. The most critical residues for the specificity of interaction are shown as sticks; **B)** The view on the CbpA^Jdom^ surface facing CbpM, showing sidechains of all residues interacting with CbpM; **C)** The conserved hydrophobic patch on CbpM surface (cyan, Leu59, Leu63, Ala71, Val72, Leu76 and Leu77) at the interface with CbpA^Jdom^ and surrounded by an acidic patch (red, Glu22, Glu62, Glu64, Asp66 and Glu79). CbpA^Jdom^ is shown as a cartoon colored beige with seven basic residues from (Lys19, Lys22, Arg26, Arg27, Arg30, His33 and Lys38) shown in stick mode.

Each CbpA^Jdom^ binds the CbpM dimer at the crevice between the two subunits, making contact with both subunits, and the contacting region is contiguous on the CbpM dimer surface. The crystal structure reveals extensive interactions between CbpA^Jdom^ and CbpM involving sixteen residues from CbpA^Jdom^ and twenty residues from CbpM. The conserved J-domain residues that were shown to be essential for DnaK binding, including His33 and Asp35 from the conserved H-P-D motif [33], as well as the neighboring residue Val36, are located in the center of the interface and interact with both molecules of CbpM (Fig. 2A). Helix α2 of CbpA^Jdom^ provides the majority of the interacting residues, with additional contributions from the loop α2/α3 and helix α3 (Fig. 2A).

The interface on CbpM involves residues mainly from helices α2 and α4 of one dimer subunit and helices a3′ and a4′ of the other subunit. Since almost half of the residues of CbpM contacting CbpA^Jdom^ are located on helix α4, the coiled-coil region not only contributes to the dimerization of CbpM, but also plays a critical role in binding to CbpA^Jdom^ (Fig. 2A). We have previously shown using mutagenesis that residues Tyr25, Arg26, Ala29, His33, Val36 and Phe46 of CbpA^Jdom^ are important for CbpM binding [22]. The crystal structure of the complex shows that these residues are all directly involved in contacting CbpM either through hydrogen bonds or van der Waals interactions (Fig. 2A). Additional ten residues of CbpA^Jdom^ (Lys19, Lys22-Ala24, Arg27, Arg30, Pro34-Asp35, Lys38 and Trp53; Fig. 2B) participate in the interface, proving it to be much more extensive than originally anticipated [22]. In CbpM, a highly conserved patch of hydrophobic residues (Leu59, Leu63, Ala71, Val72, Leu76 and Leu77) constitutes a central region of the interface and is surrounded by much less conserved polar residues (Fig. 2C). The only highly conserved CbpM polar residue facing CbpA is Glu62. This residue forms a hydrogen bond with His33^CbpA^ of the conserved H-P-D sequence motif and was recently shown to be essential for CbpA binding [18]. In addition to Glu62, the CbpM interface involves four other acidic residues (Glu22, Glu64, Asp66 and Glu79) while CbpA^Jdom^ contributes seven basic residues (Lys19, Lys22, Arg26, Arg27, Arg30, His33 and Lys38) (Fig. 2C). This asymmetric charge distribution contributes to the initial electrostatic attraction driving complex formation. The tips of the acidic sidechains of CbpM residues involved in CbpA binding are solvent exposed, while their hydrophobic parts participate in van der Waals interactions with β- and γ-CH_2_ moieties on CbpA. This architecture of CbpM explains why many polar residues on the interface are generally maintaining their polar characteristics but are not otherwise conserved in γ-protobacteria.

### Regulation of the DnaK ATPase Activity by CbpM

**Figure 3:**
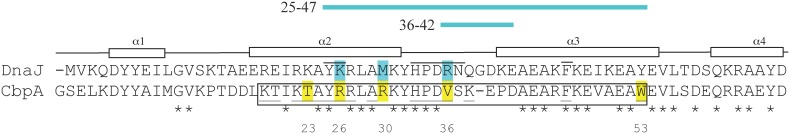
Sequence alignment of J-domains of DnaJ and CbpA. The amino acids mutated are highlighted in gray for DnaJ. The CbpA regions inserted in chimeric DnaJ are shown schematically in gray above the sequences. The CbpA region involved in CbpM binding is boxed, with residues on the interface underlined. DnaJ residues interacting with DnaK are overlined. Secondary structure elements are indicated above the sequences.

**Figure 4:**
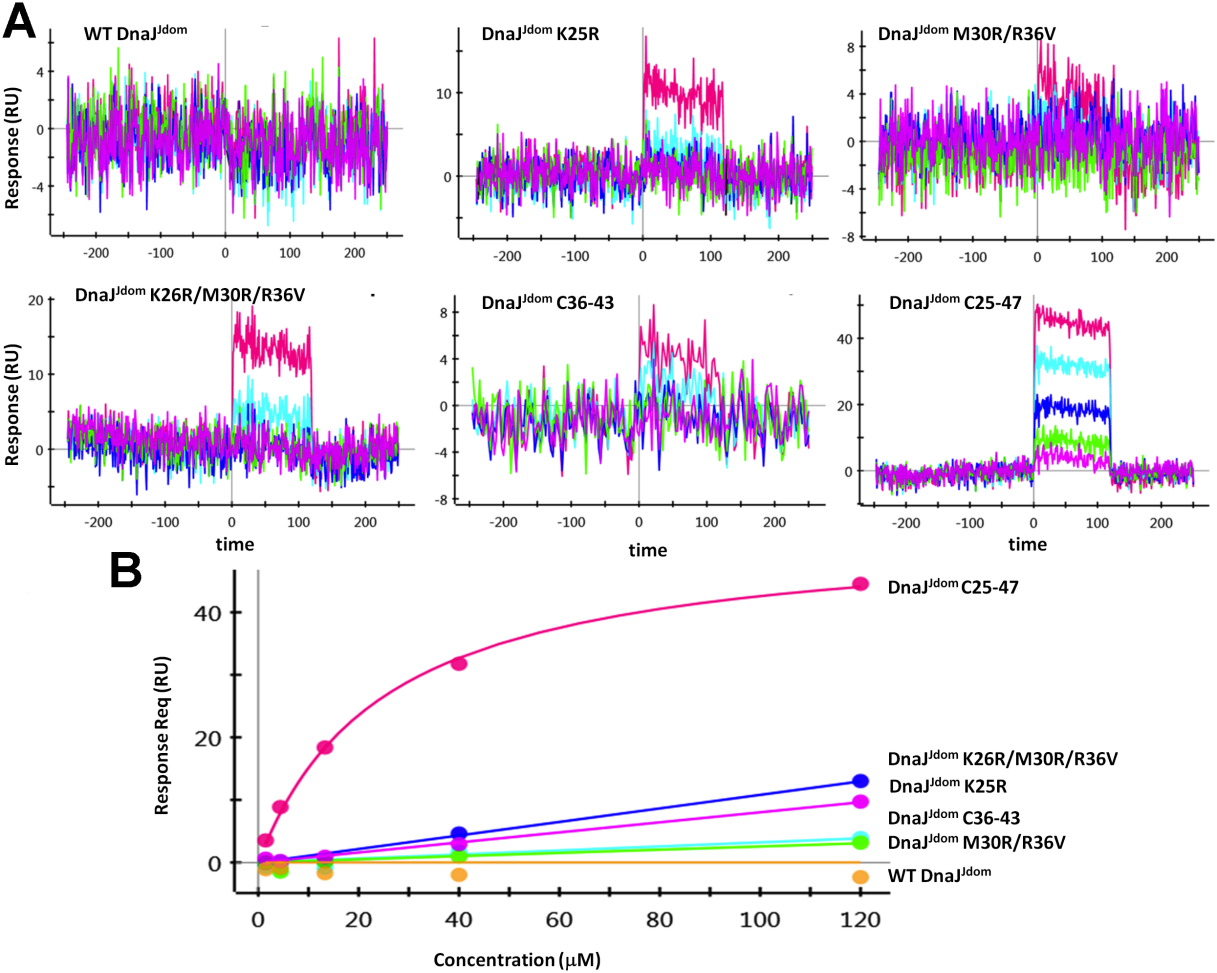
Characterization of the interaction between CbpM and engineered variants of DnaJ^Jdom^ by Surface Plasmon Resonance. **A)** Representative sensorgrams for binding of WT DnaJ^Jdom^ and the mutants to immobilized CbpM. All mutants have DnaJ residues replaced by structurally equivalent residues of CbpA. DnaJ^Jdom^(25–47) denotes a chimeric protein (25YKRLAMKYHPDRNQGDKEAEAKF47–>25YRRLARKYHPDVSKEPDAEARF46) with an incorporated central region of the CbpA^Jdom^ that binds to CbpM into DnaJ^Jdom^. DnaJ^Jdom^(36–42) represents a chimera (36RNQGDKE42–>36VSKEPD41), in which the loop between helices α2 and α3 in DnaJ^Jdom^ was replaced with equivalent loop in CbpA^Jdom^. The steady state assay was used to analyze the results and double referencing was used to subtract the buffer effects; **B)** The binding isotherms. Clearly, WT DnaJ^Jdom^ does not bind to CbpM, but all mutants containing CbpA residues show some binding. The chimera DnaJ^Jdom^ (25–47) has the highest affinity (K_D_ = 26 µM).

The structure of the CbpA^Jdom^-CbpM complex allowed us to propose the key residues that define the specificity of the J-domain for CbpM. To test this hypothesis we selected the J-domain of DnaJ, which shares 38% of amino acids with J-domain of CbpA and yet does not bind CbpM. We mutated these key residues of DnaJ to match those of CbpA and tested its binding to CbpM. In the first DnaJ construct we have replaced the entire central region of DnaJ^Jdom^ for that of CbpA^Jdom^ (^25^YKRLAMKYHPDRNQGDKEAEAKF^47^-^25^YRRLARKYHPDVSKEPDAEARF^46^) (Fig. 3). In the second construct only the loop between helices α2 and α3 was replaced (^36^RNQGDKE^42^-^36^VSKEPD^41^). The final construct had only three mutations (K26R/M30R/R36V). The DnaJ constructs were expressed and their binding to CbpM was evaluated by Surface Plasmon Resonance (SPR) experiments. Wild-type DnaJ^Jdom^ clearly does not bind to CbpM. However the chimeric DnaJ^Jdom^ with the amino acid fragment Y25-F47, representing the central region of the interface, replaced with the corresponding region of CbpA^Jdom^, binds CbpM with K_D_ of 26 µM (Fig. 4). The second DnaJ construct with replacement of loop 36–41 binds CbpM more weakly (Fig. 4). Finally, even the triple DnaJ^Jdom^ mutant shows weak binding to CbpM (Fig. 4). Similarly to what was previously observed for CbpA^Jdom^ [22], DnaJ^Jdom^ mutants bind CbpM with fast on and off rates.

**Figure 5:**
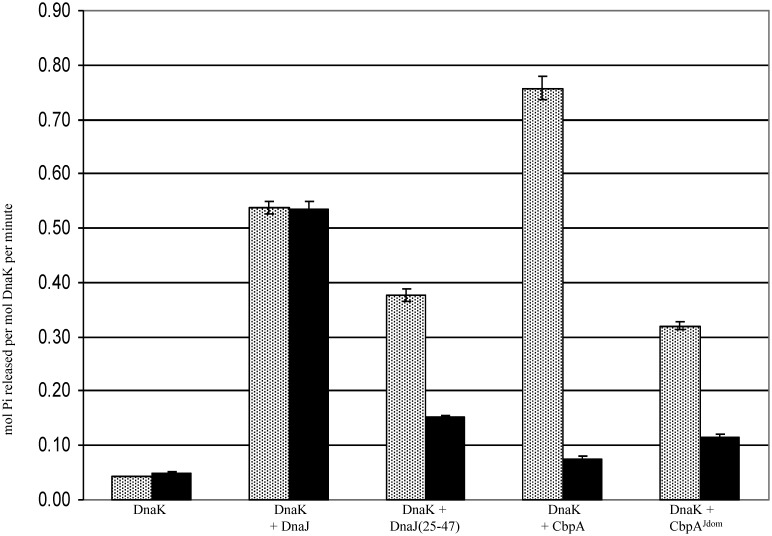
ATPase activity of DnaK measured alone, with DnaJ (1 µM), with DnaJ(25–47) (1 µM), with CbpA (1 µM) or with CbpA^Jdom^ (2 µM) in the absence (gray) and presence (black) of CbpM (4 µM). Reactions contained 0.5 µM DnaK, 0.2 µM GrpE (Assay Designs), 20 mM Tris pH 8, 75 mM KCl, 0.9 mM MgCl_2_, 38 mM NaCl and 0.5 mM ATP. After 90 minutes at room temperature free phosphate levels were measured using malachite green based dye (Innova Bioscience). Standard error was calculated from 3 experiments.

Additionally, functional assays were used to show that the interface in the CbpM-CbpA^Jdom^ complex is important in regulating chaperone activity. This regulatory activity was detected through assays measuring ATPase activity of DnaK (Fig. 5). As expected, the full-length CbpA and full-length DnaJ co-chaperones up-regulate DnaK ATPase activity. The J-domain of CbpA also stimulates ATPase activity of DnaK, however higher concentrations are required for similar potency as the full-length CbpA. Our data confirm that CbpM decreases stimulatory activity of CbpA but has no effect on activity of DnaJ. CbpM regulates CbpA^Jdom^ in a similar way to the full-length CbpA.

To further show that the interface between the J-domain and CbpM has important functional role in controlling chaperone function, we tested the responsiveness of the full-length DnaJ(Y25-F46) chimera to the presence of CbpM using the DnaK ATPase assay. To this end, the sequence Y25-F47 in DnaJ was replaced with the corresponding fragment from CbpA and assayed in conjunction with DnaK and CbpM. The assay confirmed that the chimeric DnaJ becomes regulated by CbpM (Fig. 5). Thus, the CbpM-binding interface on CbpA^Jdom^ is functionally important and provides differential specificity of CbpM for CbpA, and not DnaJ.

## Discussion

### Dimers vs. Monomers

We have shown previously using size-exclusion chromatography (SEC) that CbpM (11.5 kDa protein) elutes with apparent MW of ∼36 kDa, corresponding either to a globular trimer or to an elongated dimer [22]. The oligomeric state was also suggested in earlier studies [15] based on a broad SEC peak corresponding to 15–60 kDa. Although in the cross-linking experiments [15] the majority of CbpM treated with crosslinking agent migrated in the SDS-PAGE with an apparent molecular mass of ∼8 kDa; a minor species with an apparent molecular mass of 27 kDa (dimeric CbpM) was also present [13]. The crystal structure of CbpM sugests why these cross-linking experiments were not effective at detecting its oligomeric state. The reagent used in these experiments targets mainly side chains of lysines and N-terminal amino groups. *E coli* CbpM contains only one lysine, Lys84 (Thr84 in *Klebsiella* CbpM in the C-terminal coiled-coil (Gly69-Leu98) segment. This lysine faces Leu83′ of the second CbpM molecule and its Lys84′ is located on the opposite side of the coiled-coil, making it difficult to bridge by the crosslinker. The N-terminal amino groups of the dimer are far from each other and from Lys84. Therefore, low efficiency of cross-linking is not surprising The large interface area of ∼960 Å^2^ for each CbpM subunit strongly support predominance in solution of CbpM dimers although equilibrium between the monomers and dimers cannot be excluded.

### Biological Implications Derived from the Structure of the CbpM-CbpA^jdom^ Complex

The structure of the CbpM-CbpA^Jdom^ complex supports a competitive model of regulation of CbpA by CbpM, in which CbpA binds to DnaK or to CbpM in a mutually exclusive fashion. Although structural data for a DnaK-CbpA complex are not available yet, high sequence similarity between J-domains in co-chaperones allows using a better characterized DnaK/DnaJ system as a model for interpreting our results. The J-domain residues critical for co-chaperone activity and binding to DnaK include Tyr25, Arg26, His33-Pro34-Asp35-Arg36-Asn37 and Phe47 [33] (DnaJ numbering). Since most of these residues are conserved between the J-domains of CbpA and DnaJ, the two co-chaperones most likely bind DnaK in the same fashion, using the same surfaces as an interface. The structure of the CbpM-CbpA^Jdom^ complex reveals that nearly all of the CbpA residues expected to participate in DnaK binding are also involved in binding to CbpM. The importance of this region in CbpA for modulation by CbpM was evaluated through site-directed mutagenesis of DnaJ, focusing on residues, which are different in DnaJ and CbpA (Fig. 3), including amino acids Arg26, Arg30, Val36 and the loop between helices α2 and α3. Interestingly, even though the wild-type DnaJ^J-dom^ does not bind CbpM, mutations of these residues to their CbpA equivalents, induces weak interactions. The chimeric DnaJ, in which residues 25–47 were replaced by equivalent residues in CbpA that constitute the interface for CbpM, becomes responsive to CbpM in ATPase assays. Thus, the specificity of CbpM for CbpA, but not DnaJ, results from subtle sequence differences between the two J-domains. These sequence differences in the interface region between CbpA and DnaJ are conserved in γ-proteobacteria. The unique CbpA^Jdom^ residues that contribute to the specificity of recognition participate in an extensive network of interactions with CbpM. Arg26^CbpA^ is involved in hydrogen bonding with Glu64 and Glu26 of CbpM; the sidechain of Arg30^CbpA^ interacts with Thr75 and Leu30 of CbpM; finally, Val36^CbpA^ is located in a hydrophobic region in the CbpA-CbpM complex and forms van der Waals contacts with highly conserved residues Val72^CbpM^ and Leu76^CbpM^. Corresponding DnaJ residues could not support such interactions. Instead of Arg26^CbpA^ DnaJ has a lysine sidechain, which would not be able to form hydrogen bonds with both Glu64^CbpM^ and Glu26^CbpM^. A hydrophobic Val36^CbpA^ is replaced in DnaJ by an arginine. In contrast, sequence differences in this region of the J-domain have negligible influence on interactions with DnaK, as both co-chaperones stimulate ATPase activity to a similar extent (Fig. 5).

### Relationship between CbpM and MerR-like Transcription Regulators

**Figure 6:**
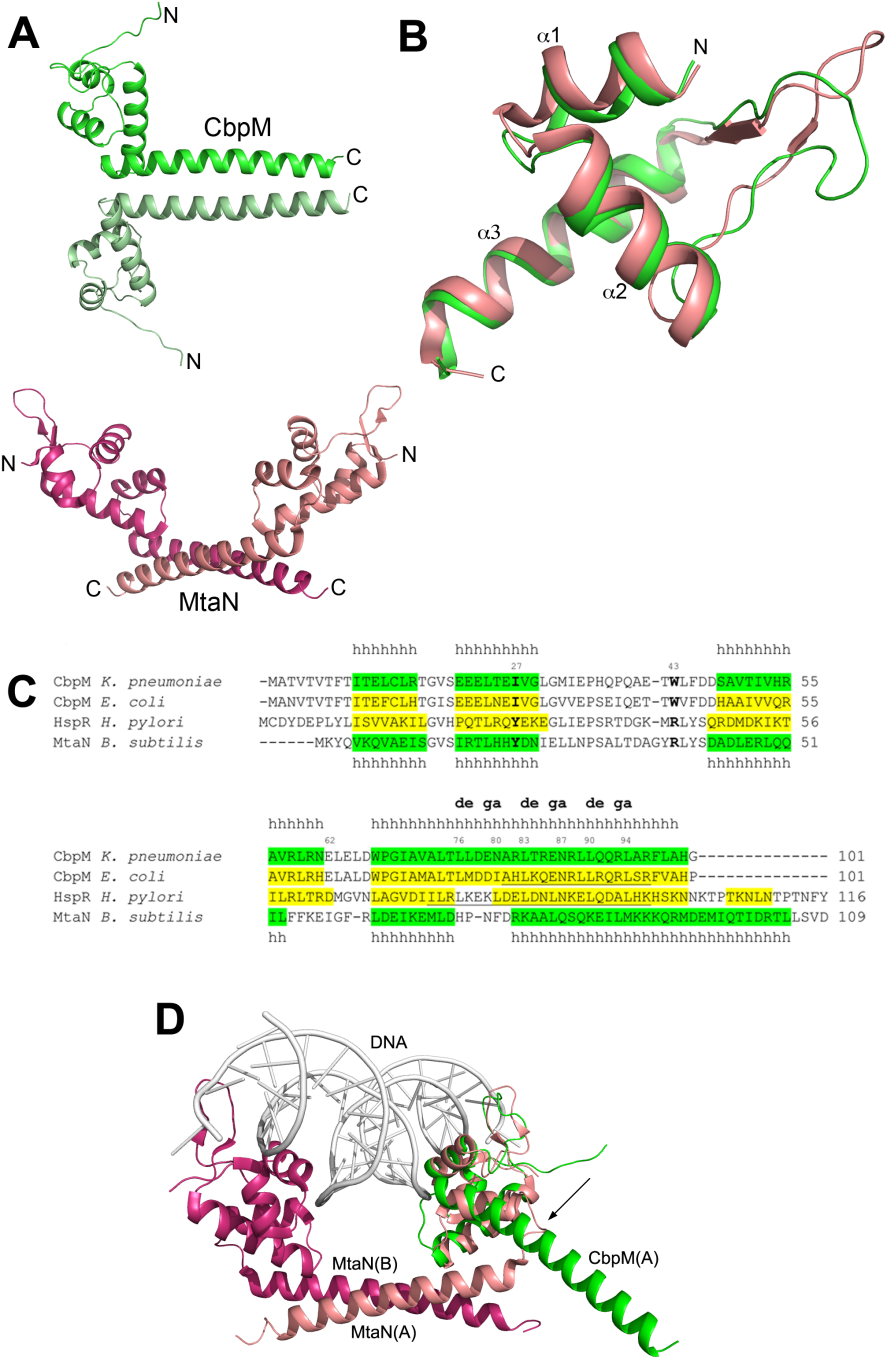
Comparison of CbpM with transcriptional activators from MerR-like family. **A)** The overall structures of the CbpM dimer (in light/lime green) and MtaN dimer (in salmon/magenta) with different orientation of the coiled-coils; **B)** Superposition of the first three helices of CbpM (in green) with MtaN (in salmon; PDB code 1R8D); **C)** Sequence alignment of CbpM from *K. pneumonia, E. coli* and HspR from *H. pylori*, along with the structure based alignment with the first 60 residues of the transcriptional regulator MtaN from *Bacillus Subtilis* (PDB code 1R8D). The residues forming helices are shown in green (experimental) with letter *h* above/below or in yellow (predicted). The coiled-coil location in *E. coli* CbpM and *H. pylori* HspR as predicted by Coil program [42] is indicated by underlined sequence. The conserved residues in CbpM, which are different from the DNA binding residues in transcriptional regulators, are shown in bold; **D)** Mechanistic model explaining how HspR (salmon/magenta) and CbpM (green) structures could evolve from each other. An arrow indicates the position where the two structures diverge. For simplicity, only one monomer of CbpM is shown. DNA bound to MtaN is shown in light gray.

The structure of CbpM reveals a striking similarity to the members of the MerR family of transcription regulators. Proteins from this family are usually involved in regulation of transcription in response to a variety of stresses [1], [34]. They have a common architecture, with an N-terminal DNA-binding helix-turn-helix domain followed by a coiled-coil region and a C-terminal effector-binding domain. The two most similar structures to CbpM, as identified by Dali [35] are MtaN from *Bacillus Subtilis* (PDB code 1R8D) and CueR from *E. coli* (PDB code 1Q06). Both CbpM and MerR-like regulators form dimers associated through their coiled-coil segments, however, while in CbpM the coiled-coils are parallel, in all the known structures of the MerR family regulators the coiled-coils are antiparallel, leading to a completely different orientation of the N-terminal globular domains within respective dimers (Fig. 6A). An antiparallel topology plays a key role in the function of transcription regulators [36], [37].

The region of the highest similarity between CbpM and MerR-like regulators comprises the N-terminal ∼70 residues, corresponding to helices α1–α3 in CbpM. Superposition of the structures for this region results in an RMSD of ∼0.7 Å for the backbone atoms (Fig. 6B). The C-terminal helix α4 in CbpM that provides coiled-coil dimerization interface has a counterpart in MerR-like transcriptional regulators that comprises a short helix pointing in the same direction as α4 in CbpM, as well as a longer helix nearly at a right angle to the first. This last helix in transcriptional regulators is also involved in a coiled-coil dimerization (Fig. 6A).

Sequence analysis clearly supports a relationship between CbpM and MerR-like transcriptional regulators, as numerous related sequences can be identified using PSI-BLAST with the *E. coli* CbpM sequence as bait, with scores of ∼10^−4^. Interestingly, the highest sequence identity is with HspR, involved in transcriptional regulation of heat shock operons, including the DnaK operon [38], [39]. There is as yet no structural data for a representative of this subgroup; nevertheless, secondary structure predictions indicate similarity to both CbpM and MerR (Fig. 6C). HspR transcription regulators are present in the same operon as CbpA (ε-proteobacteria) or DnaJ (actinobacteria) in a mutually exclusive fashion. All these arguments suggest evolutionary relationship between these two classes of proteins. Additional strong support is provided by recent results indicating that *Helicobacter pylori* CbpA can bind to HspR [17].

Comparing the structure of CbpM with MerR-like transcription regulators allows us to propose a model for their evolutionary transformation. The MerR fold can be converted to the CbpM fold by straightening the kink between helices α4 and α5 of the transcription regulator (Fig. 6D), which would combine them into one long helix similar to α4 of CbpM. MerR transcription regulators bind DNA through their helical N-terminal domains [38]. While the overall structure of this region in CbpM is very similar, CbpM does not have residues necessary for binding DNA that are conserved in transcription regulators. These residues include a tyrosine and arginine in the binding site of transcription regulators, which are replaced by Ile27 and Trp43 in CbpM.

Both structural and functional link between transcription and chaperone regulation provides a new perspective and may shed new light on the poorly understood role of DNA binding by CbpA. Binding of CbpA to intrinsically curved DNA [13], which is frequently found in promoter regions [40], may be relevant to its involvement in transcription regulation [18]. In *Streptomyces coelicolor* DnaK acts as a transcriptional co-repressor, forming stable ternary complexes with HspR and DNA [41]. In *H. pylori*, binding of the co-chaperone CbpA to HspR has an opposite functional effect [15]. Clearly, formation of dynamic complexes between DnaK, CbpA, HspR (CbpM) and DNA plays an important role in stress regulation. The unique structure of the CbpM-CbpA^Jdom^ complex provides a basis for further functional studies of these systems.
